# Building a CTU Orientation Handbook iPad^®^ application for first-year residents

**Published:** 2019-03-13

**Authors:** James Chan, Fan Yang, Babak Rashidi, Isabelle Desjardins, Di Maria Jiang

**Affiliations:** 1 Faculty of Medicine, University of Ottawa, Ontario, Canada; 2 Department of Family and Community Medicine, University of Toronto, Ontario, Canada; 3 Faculty of Medicine, University of Ottawa, Ontario, Canada; 4 Divison of Medical Oncology, Department of Medicine, University of Toronto, Ontario, Canada

## Abstract

**Background:**

The General Internal Medicine Clinical Teaching Unit (CTU) is a challenging rotation for new residents and the optimal format of orientation has not been determined. We hypothesized that an iPad^®^ application (app) would be a useful reference tool after residents completed their traditional large group orientation.

**Methods:**

Postgraduate year 1 (PGY1) residents were sent a link to download the free app one week before the start of their rotation. A pre-usage survey at initial login collected basic demographics. Usage data was collected to determine the sections, duration, and the timeframe from which the app was utilized.

**Results:**

Pre-usage survey data revealed that 63% of participants were female, 69% felt the app would improve orientation, and 94% were comfortable using mobile technology for medical education. Usage data showed “Teaching Sessions and Schedules,” “The Consult Note,” and “Admission Orders” were the three sections most commonly used. The most usage was during the evening call shift (10pm to 6am), followed by the morning shift (6am to 5pm).

**Conclusion:**

The CTU Orientation App was a useful supplement to the traditional orientation. Researchers may not be able to predict what content would be most valuable in an iPad^®^ app, thus pre-development needs-assessments and usage feedback are crucial.

## Introduction

The General Internal Medicine Clinical Teaching Unit (CTU) is known to be a challenging rotation because of high patient load, high acuity, and complexity of patients. At the Ottawa Hospital, the CTU orientation is a one-hour PowerPoint^®^ lecture that is presented at the beginning of each rotation to all trainees by chief internal medicine residents. Many topics are covered in that time frame such as clinical responsibilities, call schedules, rotational objectives, and trainee question period. Our rotational evaluations indicate residents find their CTU orientation overwhelming. Lack of standardization, flexibility, accessibility, and information overload prevent effective orientations.^1^^-^^3^ An ideal orientation should be comprehensive, focused on practical issues, frequently updated, standardized, and accessible regardless of time and place. Other modalities have also been explored, including online modules and videos,^2^ simulations, mock clinical scenarios,^4^ multiple choice sessions,^5^ and web-based clinical orientations^3^, but they too fall short.

**Figure B1:**
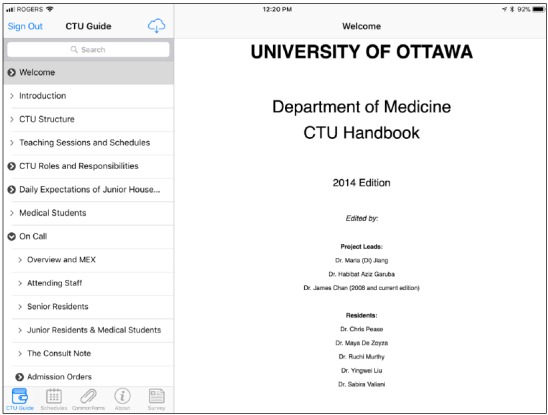
Screenshot of CTU Handbook App

Since all trainees at our institution have access to the iPad^®^ for clinical duties, we created an iPad^®^ app to supplement their orientation (Figure B1). Studies have shown technologically-savvy residents benefit from apps in medical education.^6^^-^^9^ Although research in the area of technology and orientation is nascent, it has been demonstrated that learners perceive the integration of mobile technology will potentially improve their knowledge acquisition, facilitate collaborations, and enhance their efficiency,^10^^,^^11^ However some authors have warned of the distraction and privacy issues.^12^ In this paper we report the usage trends for this app, including anticipated versus actual app utilization. We will also discuss the impact of this study on future orientation app development, and for medical education in general.

## Methods

Ethics approval was obtained from the Ottawa Health Science Network Research Ethics Board on April 14, 2015 (Protocol #20150130-01H).

### App development

Local content experts used input from the pre-existing paper-based orientation binder as well as from members in the training committee and the internal medicine residency program director to determine content (Appendix A) included in the app. They targeted the app towards Year 1 residents who were rotating through the CTU for the first time. A pre-usage survey was built into the app to collect basic demographics and identify baseline familiarity with mobile technology in medical education (see Appendix B). Once testing was complete, the app was made available as a free download in iTunes^®^. The app requires an internet connection to refresh its content and the hospital has campus-wide Wi-Fi access. The Internal Medicine Training Committee and the Division of General Internal Medicine jointly funded the app development.

### Research participants

The postgraduate medical program is organized into 13 rotations. Thus, rotation 1 represents the beginning and rotation 13 represents the end of the academic year. Participants were University of Ottawa PGY1 residents regardless of training program scheduled on CTU between rotation 12 to rotation 4 (6 clinical rotations).

### Usage data

The software developer remitted the participants’ anonymized usage data for the date range requested to be analyzed.

## Results

### Survey results and demographics

Sixty-seven residents consented to participate within the study period, but we were only able to collect 35 pre-usage surveys. Overall, 63% of research participants were female, and 84% of users were between 25 to 29 years of age.

Of the 35 users surveyed, 33/35 (94%) reported being comfortable to very comfortable with using mobile technology, and 74% felt the app could improve the orientation experience. Participants anticipated that the app could help them with the admission algorithm, formulary, clinical guidelines, consultation rule and procedures, and call schedule, but there was no consensus on the section of the app they anticipated being the most commonly used.

### Usage data

Microsoft Excel^®^ was used for all descriptive statistics. The app was accessed 909 times by 41 participants with an average length of 78 seconds (Table 1: Analysis of Most Used Page.) However, some pages were used for a long time, but not frequently, while some pages were accessed frequently but only for a short time. To get a more accurate measure of usage, we created a “Popularity Score” defined as “minutes x frequency.”

**Table 1:** Analysis of most used page

	Total minutes	Frequency of use	Popularity score
Admission Orders	124.2	26	3229.2
The Consult Note	197.2	32	6310.4
Teaching Sessions and Schedules	58.6	47	2754.2
Junior Residents and Medical Students (on Call)	87.9	31	2724.9

The top four topics by Popularity Score were:

1. “The Consult Note” (the necessary parts of creating a consult note while on call)
2. “Admission Orders”
3. “Teaching Sessions and Schedules” (teaching sessions for the day)
4. “Junior Residents and Medical Students (on Call)” (responsibilities of a resident or medical student on call, including rounding at night)

We also grouped certain topics into themes based on conceptually similar topics (Table 2); the most common themes were:

**Table 2:** Analysis of most used sections according to theme

Thematic grouping	Total minutes	Frequency of use	Popularity score
On Call *- Overview, attending staff, consult note, admission orders*	470.6	148	69648.8
Daily expectations house staff *- medical reconciliation, eVitals, ordering tests & consults, transferring patients, handover, discharge process*	112.2	171	19186.2
Common Forms *- admission slip, echo and cardiac diagnostic requisition, leave of absence form, CHF gap tool, social work referral*	81.7	135	11029.5
Admissions - *Admission Orders, AMA admission orders, ED Admission Algorithm, Family Medicine Admitting Physicians*	140.7	54	7597.8

1. “On call”
2. “Daily expectations of junior house staff”
3. “Common forms”

### Usage by time of day

We divided usage times into three sections based on the typical working day of our CTU rotation. In our academic CTU, the PGY1 residents are on-call in shifts, with the first senior resident working from 8am to 5pm. The next shifts are 5pm-12am and 12am-8am. The app was most used from 12am to 8am (46%), followed by 8am to 5pm (30%), and least used from 5pm to 12am (24%).

### Usage by resident program

Only 8/35 participants identified themselves as internal medicine residents and 18/35 identified themselves as off-service residents; however, the app was utilized more frequently by internal medicine residents, as shown in Table 3. Males accessed more sections (23.1 sections vs. 19.0 sections), and on more days (5.8 days vs. 3.8 days) than females, but their usage durations were similar (19.4 minutes vs. 19.8 minutes in females).

**Table 3:** Comparison of app use between core internal medicine vs. off-service residents

Type of resident	**Minutes used per resident**	**Number of pages visited per resident**	**Unique number of days used per person**
Internal medicine	32.9	35.5	8.1
Off-service	15.9	15.7	3.0

### Usage by Rotation

The study period spanned the end of one academic year (Rotation 12 & 13) and the beginning of another year (Rotation 1 through 4). Usage increased from rotation 12 until it peaked at rotation 1, thereafter dropping off dramatically (see Table 4).

**Table 4:** Usage by Rotation

Rotation	Total minutes	Number of users	Average minutes per user
12	101.23	10	10.12
13	311.58	14	22.25
1	537.53	14	38.39
2	89.78	14	6.41
3	43.91	9	4.88
4	31.53	5	6.3

### Usage by comfort with technology

**Figure 1:**
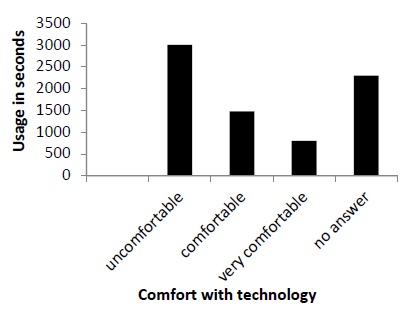
Usage trends based on comfort with technology

We found an inverse relationship between residents’ comfort level with mobile technology utilization and actual usage (Figure 1).

## Discussion

Our study looked at the utility of an iPad as a supplement to the traditional orientation during an internal medicine rotation at an academic centre. Despite technical challenges, our results showed that this app was well used and could provide a useful supplement to traditional orientation. As we expected, the CTU Handbook App was used most often at the beginning of the academic year (July), when the first cohort of PGY-1 residents were just coming onto CTU for the first time. This suggests that orientation needs do not remain static over the course of the year. Also, as expected, the app was used most frequently by residents overnight when supportive resources were more scarce and work load may be the highest. The most frequently used sections (“on-call duties,” “admissions and discharge protocols,” “the consult note,” and “common forms”) suggest that it is during on-call hours that residents need the most support. More research is needed in this area.

We had anticipated that certain sections, such as the On-Call Schedules (Popularity Score 889.2), Medical Reconciliation (Popularity Score 169.2), and Drug Formulary (Popularity Score 652.8) would be most frequently accessed; this was not the case, and there could be several reasons. Firstly, the on-call schedule was also available through emails, making the content redundant. Secondly, while the Medical reconciliation was a relatively new and complicated process for junior residents, the instructions to use it was not frequently accessed in the app. Awareness may be insufficient, and residents would favour trial and error rather than reading lengthy instructions. Finally, the drug formulary was also available within the hospital intranet, and it was a rather large pdf document to load on the app. We felt these challenges highlighted that, sometimes, even medical education experts can’t reliably anticipate what residents might need while on call. The more prudent way to proceed before the app development process may have been a formal needs assessment with the users.^12^ This also highlights the need for ongoing post-app usage and feedback tracking, to allow for continuous improvement of the app after its initial rollout. It is essential to capture patterns of app usage to improve appropriate delivery of information on mobile app platforms.^13^

Interestingly, we found that residents who were most comfortable with technology tended to spend less time on the app, and those who reported that they were less comfortable spent less time on the app (Figure 1). We speculate that this is because technology-savvy residents need less time to navigate, but it may also be that they scan information more and read less.^14^ They may also have the ability to navigate other overlapping hospital resources to retrieve information quickly. This is an area where further research could provide insight.

### Challenges and lessons learned

One of the main challenges was the availability of technical support. Many residents experienced login and registration difficulties, which required quick resolution with technicians. This may have led to some user dissatisfaction or early disengagement for some users. Additionally, necessary content updates were frequent after the app launch, making timely technical support necessary to keep information current.

Data collection was also a challenge. Although the pre-usage survey was mandatory prior to the app usage, we only received 35/41 surveys. Part of this could be explained by the fact that not all of the participants who actually consented used the app. Although we received usage data from 41 users, some participants were able to bypass the survey. After extensive investigations we could not determine how users could have bypassed the survey.

Users also did not provide detailed comments in the short answer sections of the survey, and we feel that better question design may have helped increase response. We had planned post-usage focus groups, however due to difficulties in recruiting residents after their rotations, this was not completed.

Our usage data tracking methods were not granular enough to yield usage data with sufficient precision to support more detailed analysis. For instance, if a user stayed on one page while leaving the iPad idle, the data will over-emphasize the usage of that particular page. To overcome this issue, we suggest that tracking actual user interaction may be superior to tracking simple time per page. Additionally, researchers can incorporate “search tracking” that records what information users were looking for.

### Conclusion

The process of designing, building, and implementing the CTU Orientation Handbook iPad^®^ app is feasible, practical, and may be a useful supplement to the traditional orientation.

When developing apps for medical education, developers and content experts are not always able to predict what features or contents will be the most useful, partly due to lack of understanding and evidence. Currently available tools only demonstrate moderate ability for predicting usage patterns of medical education apps.^15^ Therefore, formal needs assessments and subsequent content updates following release, are likely to result in a more useful and sustainable app. Lastly, it may be helpful to use additional mechanisms to understand the needs from users, such as usage and search tracking for ongoing app improvement.

## Appendix A

**Summary of Final Headings of the CTU Handbook application**

Welcome
Overview
Usage of Data
iMedMedia
Introduction
CTU Structure
Teaching Sessions and Schedules
CTU Roles and Responsibilities
Attending Physicians
  Senior Medical Residents
  Junior Medical Residents
  Medical Students
Daily Expectations of Junior Housestaff
  Overview
  Medical Reconciliation (MedRec)
  eVital Signs
  Ordering Labs
  Ordering Diagnostic Imaging
  Consulting Other Services
  Transferring out/into AMA (Acute Monitoring Area)
  Weekend/End of Rotation Handover
  The Discharge Process
  Communications to Patient and Family
  Tips for Success
Work Rounds
Discharge Planning
  Allied Health
  Discharge Destinations
  Discharge Preparation Checklist
  Discharge Summaries
  Follow Up of Discharged Patients
On Call
  Overview
  Attending Staff
  Senior Residents
  Junior Residents and Medical Students
  The Consult Note
  Admission Orders
The RACE Team
Consult and Triage Team
AMA (Acute Monitoring Area)
Procedures on CTU
Educational Goals and Objectives
  PGY1 Residents
  PGY2 Residents: Non-Medicine Residents
  PGY2 Residents: Medicine Senior Residents
  PGY2/3 Residents: Night Float/Medicine Consults
Med Student (MS3) CTU Objectives
Disclaimers
appendices
Dangerous Abbreviations and Expressions
(B1) DNR Policy and Code Status
(B2) Reference for TOH Policy on CPR and Code Status
Pronouncing Death
Criteria for Step Down Units
(E1) ED Admission Algorhythm
(E2) Family Medicine Admitting Physicians
Drug Formulary
Program Descriptions for GAU, STR, TCU
GAU (Geriatric Assessment Unit)
Allied Health Contracts
Subacute Criteria Document
AMA Flow Chart
CCAC Referral Process
(M1) Ebola Virus
(M2) Viral Hemorrhagic Fever
TOH UTI Pathway
UTI - asymptomatic Bacturia
UTI - Ca associated
UTI - systemic
UTI - without systemic
Common Forms
Warfarin Dosage Protocol
Suspected/Confirmed DVT/PE
Unfractionated Heparin Nomogram ACS
Unfractionated Heparin Nomogram VTE
Pretransfer STEMI treatment
Antifungal
Chest tube insertion
Central venous catheter
PleurX program
Indwelling urinary catheter
PICC
SC Insulin administration
Delirium
Constipation
Influenza pandemic
AMA admission orders
Code Status
Alcohol Withdrawal Syndrome
TPN
Nicotine Replacement
Physical Restraints
IV NAC
PPI for upper GI bleed
Pamidronate
Pain Management
Telemetry Form
IBD Admission Form
ID Outpatient Consult
Enteral Feeding Form
Acute Stroke Admission
Admission Slip
Consent for Blood Products
Consent for Procedures
Consult Form
Dietician Consult
Echo and Cardiac Diagnostics
EEG
EMG
Geri-Psych Referral
Leave Against Medical Advice
Leave of Absence
MRI checklist
OT referral
PFT req
Physician Order Form
Progress Note
PT referral
Release of Patient Information
Sleep Study
SLP Consult
Social Work Referral
Swallowing Screen
Wound and Ostomy Consult
CCAC Referral
CHF Gap Tool

## Appendix B

**CTU Orientation Handbook iPad^®^ app Pre-Usage Survey**

1. I am a PGY-1
  - PGY-1 (Core Internal Medicine)
  - PGY-1 (Off-service)
  - Other (specify) ___________________________
2. I am
  - Male
  - Female
3. What age group do you belong to?
  - 20 to 25
  - 25 to 30
  - 30 to 35
  - 35 to 40
  - Above 40
4. What do you anticipate will be the most used features of this CTU orientation app for you?
5. What do you anticipate is the least used features of this CTU orientation app for you?
6. Compare to your peers, where would you rank your preparedness for this CTU rotation after the traditional orientation?
  - Below Average
  - Average
  - Above Average
7. In General, how comfortable are you with using technology in your own education?
  - Uncomfortable
  - Comfortable
  - Very comfortable
8. As compared to the traditional form of orientation that you just had, how will the CTU orientation handbook app improve your orientation experience?
9. Any questions or comments?
